# Selenium-containing protein from selenium-enriched *Spirulina platensis* antagonizes oxygen glucose deprivation-induced neurotoxicity by inhibiting ROS-mediated oxidative damage through regulating MPTP opening

**DOI:** 10.1080/13880209.2021.1928715

**Published:** 2021-06-01

**Authors:** Xiaojie Song, Lijun Zhang, Xin Hui, Xiangfu Sun, Juntao Yang, Jinlei Wang, Hualian Wu, Xianjun Wang, Zuncheng Zheng, Fengyuan Che, Guojun Wang

**Affiliations:** a Department of Neurology, Guangzhou University of Chinese Medicine, Guangzhou, China; b Department of Neurology, Linyi People’s Hospital, Linyi, China; c Department of Internal Medicine, Taian Traffic Hospital, Taian, China; d CAS Key Laboratory of Tropical Marine Bio-resources and Ecology (LMB-CAS), Guangdong Key Laboratory of Marine Materia Medica (LMMM-GD), South China Sea Institute of Oceanology, Chinese Academy of Sciences, Guangzhou, China; e Department of Rehabilitation, Taian City Central Hospital, Taian, China Shandong; f Department of Neurosurgery, Taian City Central Hospital, Taian, China

**Keywords:** Ischaemic brain injury, apoptosis, neurons, mitochondrial dysfunction

## Abstract

**Context:**

Selenium-containing protein from selenium-enriched *Spirulina platensis* (Se-SP) (syn. *Arthrospira platensis* [Microcoleaceae]) showed novel antioxidant activity. However, the protective effect of Se-SP against oxygen glucose deprivation (OGD)-induced neural apoptosis has not been reported yet.

**Objective:**

To verify whether Se-SP can inhibit OGD-induced neural apoptosis and explore the underlying mechanism.

**Materials and methods:**

Primary hippocampal neurons were separated from Sprague–Dawley (SD) rats. 95% N_2_ + 5% CO_2_ were employed to establish OGD model. Neurons were treated with 5 and 10 µg/mL Se-SP under OGD condition for 6 h. Neurons without treatment were the control group. Neural viability and apoptosis were detected by MTT, immunofluorescence and western blotting methods.

**Results:**

Se-SP significantly improved neuronal viability (from 57.2% to 94.5%) and inhibited apoptosis in OGD-treated primary neurons (from 45.6% to 6.3%), followed by improved neuronal morphology and caspases activation. Se-SP co-treatment also effectively suppressed OGD-induced DNA damage by inhibiting ROS accumulation in neurons (from 225.6% to 106.3%). Additionally, mitochondrial dysfunction was also markedly improved by Se-SP co-treatment via balancing Bcl-2 family expression. Moreover, inhibition of mitochondrial permeability transition pore (MPTP) by CsA (an MPTP inhibitor) dramatically attenuated OGD-induced ROS generation (from 100% to 56.2%), oxidative damage, mitochondrial membrane potential (MPP) loss (from 7.5% to 44.3%), and eventually reversed the neuronal toxicity and apoptosis (from 57.4% to 79.6%).

**Discussion and conclusions:**

Se-SP showed enhanced potential to inhibit OGD-induced neurotoxicity and apoptosis by inhibiting ROS-mediated oxidative damage through regulating MPTP opening, indicating that selenium-containing protein showed broad application in the chemoprevention and chemotherapy against human ischaemic brain injury.

## Introduction

Stroke is the second leading cause of death and disability in the world, and more than 80% of them are ischaemic stroke patients (Avan et al. 2019). There were more than 13 million new cases every year, and the number of death or disability has increased nearly two times from 1990 to 2016 (Collaborators GBDN 2019). However, the effective neuroprotective drugs that can be used in the clinic for the therapy of ischaemic stroke are very limited. Moreover, due to the narrow therapeutic time window, the application of recombinant tissue plasminogen activator (rt-PA) and endovascular thrombectomy is still limited (Powers et al. 2018). Hence, novel neuroprotective agents against brain ischaemic injury are urgently needed recently.

Severe stenosis or complete occlusion of cerebral blood vessels after ischaemic stroke may cause a sharp decrease in the blood supply to brain tissue (Liu and McCullough 2014). Deprivation of oxygen and glucose (OGD) will cause the degeneration and death of neurons involving multiple mechanisms (Yang et al. 2018). Studies have demonstrated that overproduction of reactive oxygen species (ROS) was detected at the stage of ischaemia and reperfusion of ischaemic stroke (Moro et al. 2005; Yang et al. 2018; Andrabi et al. 2020). ROS generation is prior to morphological changes of apoptosis, and mitochondria can be redistributed and activated to enhance ROS generation by regulating mitochondrial permeability transition pore (MPTP), which eventually results in apoptosis of neurons (Martin et al. 2011). Mitochondria as the main source of ROS can produce excessive superoxide anion (O^−2^), which is the precursor of hydrogen peroxide (H_2_O_2_) and hydroxyl radicals (–OH), through various metabolic and enzymatic reactions (Andrabi et al. 2020). ROS-mediated oxidative stress has been verified to play crucial roles in the pathogenesis of ischaemic stroke (Rodrigo et al. 2013; Andrabi et al. 2020). Overproduced ROS can damage the lipid, protein and DNA of cells by triggering multiple molecular signalling pathways, and eventually lead to neurons dysfunction, apoptosis or necrosis (Rodrigo et al. 2013). Mitochondria-dependent intrinsic apoptotic pathway can be triggered by ROS through regulating the MPTP opening, mitochondrial membrane potential (MMP), the release of cytochrome *c*, Bcl-2 family expression and enzymolysis of downstream caspases, and cause apoptotic cell death (Zhu et al. 2004; Liu et al. 2016; Farajdokht et al. 2018). Therefore, suppression of mitochondrial dysfunction and ROS generation represents an effective strategy in the therapy of ischaemic brain injury.

Selenium (Se) is an essential micronutrient for human health, which has been used in the prevention and treatment of various diseases because of its strong antioxidant, antitumor and immunoregulatory activities (Fairweather-Tait et al. 2011; Lipinski 2019). Increasing pieces of evidence have shown that Se possesses novel neuroprotective properties. It was reported that ebselen and (PhSe)_2_, two organic selenium compounds, can effectively suppress amyloid β-peptide (Aβ)-induced neurotoxicity in an *in vitro* model of Alzheimer’s disease (Godoi et al. 2013). Studies convinced that Se had the potential to improve scopolamine-induced memory impairment by inhibiting oxidative stress and mitochondria-mediated apoptosis in aged rat hippocampus and dorsal root ganglion neurons (Balaban et al. 2017). Selenocysteine (SeCys), a selenium-containing amino acid, can protect HT22 cells from OGD-induced neurotoxicity by inhibiting ROS-mediated oxidative damage and mitochondria-mediated cell death pathway (Wang et al. 2018). *Spirulina platensis* (syn. *Arthrospira platensis* [Microcoleaceae]) containing abundant nutritional substances has been widely used as health food. *Spirulina platensis* is a suitable carrier of selenium, and absorbed selenium is primarily encoded into its proteins (Chen et al. 2006; Sun et al. 2018). Research demonstrated that selenium-containing protein derived from Se-enriched *S. platensis* (Se-SP) is able to protect hepatocyte, erythrocytes and osteoblasts from oxidative stress-mediated injury and apoptosis by inhibiting mitochondrial dysfunction (Zhang et al. 2011; Fan et al. 2012; Sun et al. 2018). However, the role of Se-SP against cerebral ischaemic injury has been not explored yet. In the present study, the protective potential of Se-SP against OGD-injured primary in hippocampal neurons was investigated, and the underlying mechanism was fully explored.

## Materials and methods

### Materials

DMEM-F12 medium, phosphate buffered solution (PBS), foetal bovine serum (FBS), and DCFH-DA probe were all purchased from Invitrogen. MTT, propidium iodide (PI) and other agents were obtained from Sigma. TUNEL-DAPI kit, Mito-SOX probe and BCA assay kit were purchased from Beyotime Institute of Biotechnology (Shanghai, China). All primary antibodies were obtained from Cell Signalling Technology (Beverly, USA). All solvents used were of high-performance liquid chromatography (HPLC) grade.

### Culture of Se-enriched *S. platensis*

*Spirulina platensis* cells were cultured in 1000 mL Erlenmeyer flasks containing 500 mL Zarrouk medium under indicated conditions (28–30 °C, 4000 lx light and 14:10 h light:dark cycle). According to a previous report, Se-enriched *S. platensis* was cultured by adding selenium step by step (Fan et al. 2012). In brief, 100, 150, and 200 mg/L sodium selenite (Na_2_SeO_3_), an inorganic form of Se, was added to the culture medium on days-7, day-8, and day-9, respectively. The final accumulative concentration of Se was 450 mg/L. The morphology and fluorescent feature of *S. platensis* cells were respectively observed by phase-contrast microscope and fluorescence microscope. On day-11, *S. platensis* cells with and without Se were collected and lyophilised at −20 °C for subsequent experiments.

### Extraction and identification of Se-containing protein (Se-SP)

The extraction of Se-containing protein from Se-enriched *S. platensis* was conducted according to a previous report (Fan et al. 2012). Briefly, the freeze-dried *S. platensis* cells were maintained in 50 mM PBS (pH7.0). *Spirulina platensis* cells were repeatedly frozen and thawed 10 times at −20 °C and 4 °C, and treated with ultrasonication for 3 min. Then, the cell solution was centrifuged for 30 min at 11,000 *g*, and the supernatant fluid containing Se-SP was collected for future tests. SP and Se-SP were quantified by BCA kit according to the manufacturer’s instructions. The spectral characteristic of Se-SP was examined by a fluorescence microreader.

### Isolation and identification of primary neurons

Primary hippocampal neurons were isolated from newborn Sprague–Dawley rats in 24 h. Briefly, the hippocampus of neonatal SD rats was dissected, separated meninges, and shredded in Ca^2+^- and Mg^2+^-free Hank’s Balanced Salt Solution (HBSS). After being digested with 0.2% trypsin for 20 min at 37 °C, the neurons were filtrated and placed in 0.01% poly-L-lysine-coated 96-well plates supplemented with DMEM medium at 37 °C under a humidified atmosphere with 5% CO_2_. DMEM medium contains 50 mg/mL penicillin/streptomycin, 10% horse serum and 10% FBS. After culturing for 12 h, the medium was replaced with a neurobasal medium containing 2% B27, 1% penicillin/streptomycin and 0.5 mM glutamine. To inhibit the growth of glial cells, cytosine arabinoside (6 ng/mL) was added to the medium at 36 h. Half of the medium was replaced every two days. After 7 days of growth, mature neurons are employed in *in vitro* studies for the future. All animal experiments were approved by the Institutional Animal Ethics Committee of Taian City Central Hospital. All surgery was performed under isoflurane (30% O_2_/70% N_2_O mixture) via a facemask, and every effort was made to minimize suffering.

### Drug treatment and neuronal viability

The oxygen glucose deprivation (OGD) model was established to simulate ischaemic damage in stroke. Primary hippocampal neurons were cultured with glucose-free Earle’s solution (glucose deprivation) in the environment of 95% N_2_ and 5% CO_2_ (oxygen deprivation) for 6 h. Hippocampal neurons were cultured under OGD conditions for 1–13 h to determine OGD neurotoxicity. To detect the cytotoxicity of Se-SP or SP, neurons were treated with SP or Se-SP alone at different concentrations (5, 10, 20 μg/mL) for 48 h. For the protective experiment, primary neurons were treated with OGD or/and Se-SP or SP (5 and 10 μg/mL) for 6 h. Neurons were pre-treated with 5 μM CsA (a MPTP inhibitor) for 30 min before other treatments. Cell viability was detected by MTT assay. Cell viability was shown as a percentage of the control group.

### Caspase activity

Caspase activity was measured by specific caspase substrates. Neurons after treatment were harvested by centrifugation, dissolved and lysed in lysis buffer, and the total protein was quantified by a BCA kit. The total protein (100 μg/well) and specific caspase substrates (Ac-DEVD-*p*NA for caspase-3, Ac-IETD-*p*NA for caspase-8, and Ac-LEHD-*p*NA for caspase-9) were successively added into a 96-well plate and incubated at 37 °C for 2 h. The fluorescence was measured at a wavelength of Ex 380 nm and Em 440 nm by a fluorescent microplate reader. Caspase activity was presented as % of the control.

### TUNEL-DAPI staining

Neuronal apoptosis was examined by TUNEL-DAPI assay. Neurons after treatment were fixed with 4% paraformaldehyde at room temperature for 15 min and rinsed with PBS three times. 0.1% Triton X-100 in 1% sodium citrate was used to facilitate neuronal permeability on ice for 2 min. Then, cells were exposed to TUNEL reaction mixture for 1 h and co-stained in DAPI for 15 min at 37 °C. Images were obtained by a fluorescence microscope and TUNEL-positive cells (green fluorescence) were calculated to analyze the apoptotic neurons.

### Evaluation of mitochondrial membrane potential (MMP)

JC-1 probe was a kind of monomer emitting green fluorescence and was able to polymerize into J-aggregates emitting red fluorescence, has been widely used to detect mitochondrial membrane potential (MMP). The alteration of MMP from high (red fluorescence) to low (green fluorescence) can be visualized obviously by the change of JC-1 fluorescence. In brief, the treated neurons were subjected to JC-1 staining for 20 min at 37 °C in the dark. Then neurons were rinsed twice with PBS and imaged by fluorescence microscopy.

### Detection of ROS and superoxide anion

Intracellular ROS accumulation was monitored by 2,7-dichlorofluorescein diacetate (DCFH-DA) probe. DCFH-DA can penetrate into cells rapidly and was hydrolyzed by ROS into reduced green fluorescent polar derivative DCF. The mitochondrial superoxide anion was detected by a mitochondria-targeted superoxide indicator, Mito-SOX Red, which is a cationic derivative of dihydroethidum (DHE). Briefly, hippocampal neurons after treatment were seeded in a 96-well plate and incubated with DCFH-DA or MitoSOX Red for 20 min in dark at 37 °C. Then, the neurons were washed and imaged under a fluorescence microscope.

### Western blotting

Total protein was prepared from neurons and quantified by a BCA protein assay kit. Equal amounts of protein samples were separated by sodium dodecyl sulfate-polyacrylamide gel electrophoresis (SDS-PAGE) and electrically transferred onto a nitrocellulose membrane. Membranes were blocked with 5% non-fat milk in Tris-buffered saline and Tween-20 for 1 h at room temperature and then incubated with indicated primary antibodies at 4 °C for overnight. The membranes after washing with PBS were incubated with the relevant secondary antibodies (goat anti-mouse or goat anti-rabbit) conjugated with horseradish-peroxidase (HRP) for 1 h at room temperature. The immunoblotting protein bands were detected by enhanced chemiluminescence substrate. The expression of β-actin served as the internal reference.

### Statistical analysis

All experiments in the study were conducted 3 times. Experiment data were analyzed with SPSS 17.0 software and shown as the mean ± standard deviation (SD). One-way analysis of variance (ANOVA) test followed by Turkey *post hoc* test was performed to determine statistical comparisons among different groups. Bars with ‘*’ or ‘**’ represent *p <* 0.05 and *p <* 0.01, respectively. Bars with different letters indicate the *p <* 0.05 level.

## Results

### Identification of Se-SP and primary neurons

**Figure 1.**
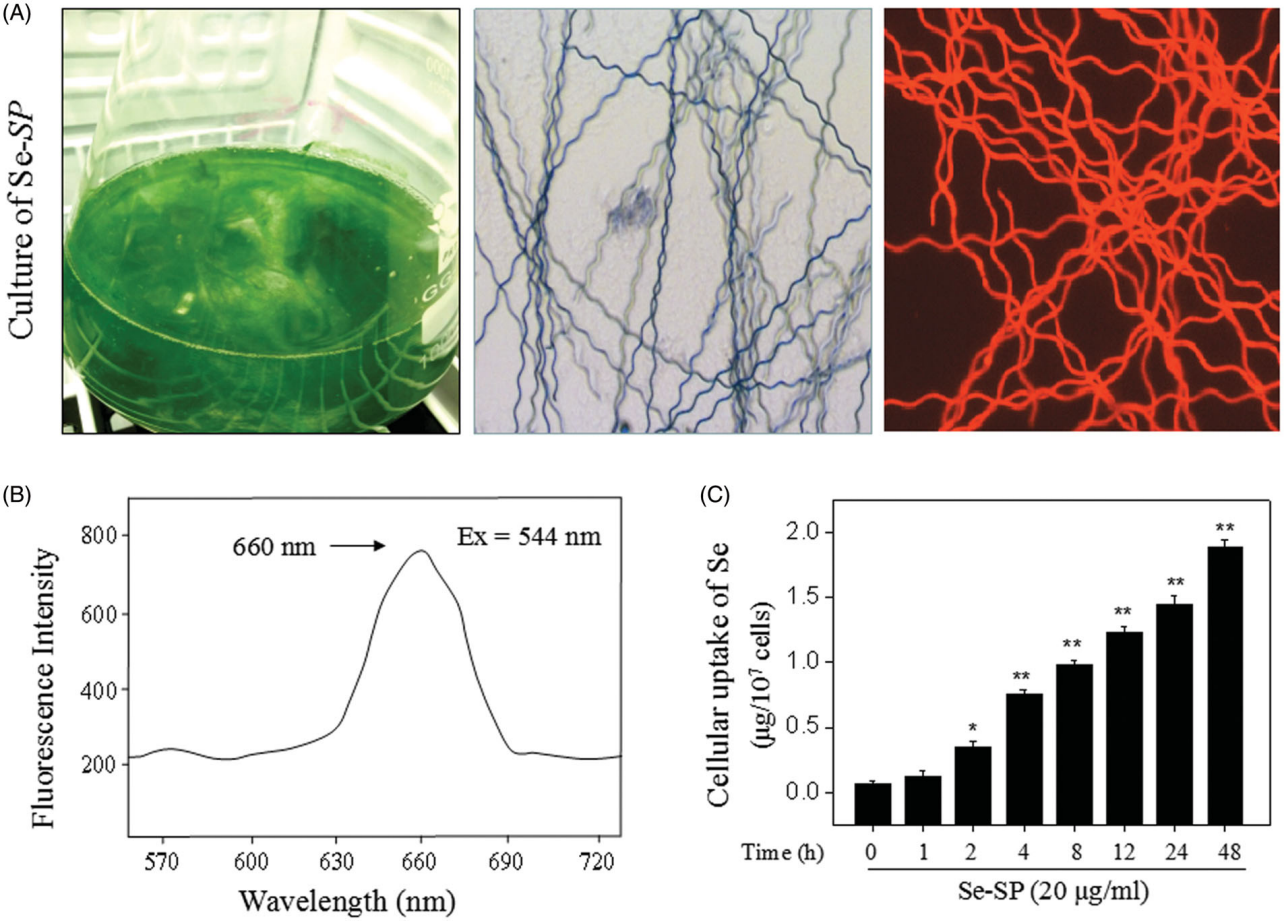
Isolation and identification of Se-SP. (A) Culture of Se-enriched *S. platensis*. Se-enriched *S. platensis* was cultured with Zarrouk medium (pH 9.0) in a 1000 mL Erlenmeyer flask. Morphology of Se-enriched *S. platensis* was detected by light microscope and fluorescence microscope. (B) Spectroscopic feature of Se-SP. The fluorescence spectrum of Se-SP crude was examined by fluorescence microreader. (C) Intracellular uptake of Se-PC. MOVAS cells were treated with 20 μg/mL Se-SP for 1–48 h, and the intracellular uptake of Se-SP was examined by ICP-AES method. All data was shown as mean ± standard deviation (SD) of three different experiments. Bars with ‘*’ or ‘**’ represent *p <* 0.05 and *p <* 0.01, respectively.

Se-enriched *S. platensis* was cultured in an Erlenmeyer flask with Zarrouk medium. Se-enriched *S. platensis* exhibited an obvious spiral appearance under the microscope with the same as that of *S. platensis* (without selenium). Se-SP also showed bright red fluorescence due to the presence of allophycocyanin (APC) (Figure 1(A)). To further identify the feature of Se-SP, a fluorescent spectrum was detected, and the results showed that Se-SP showed a significant fluorescent peak at 660 nm, which was the specific peak of APC (Figure 1(B)). Moreover, the elemental analysis revealed that Se-SP successfully permeated the cell membrane and accumulated in neurons in a time-dependent manner (Figure 1(C)).

### Se-SP inhibits OGD-induced neuronal toxicity

**Figure 2.**
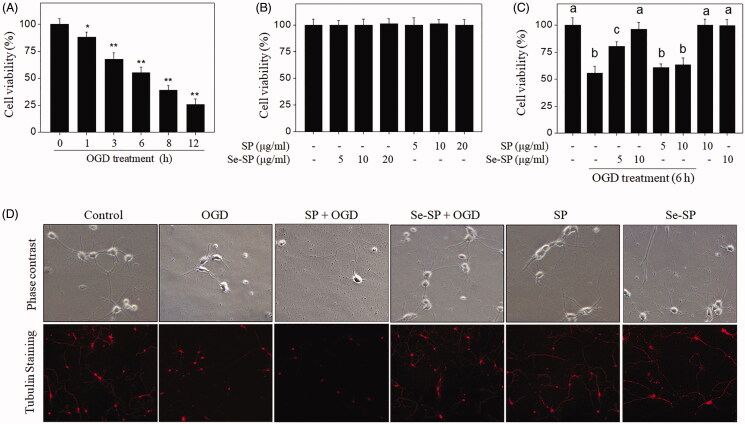
Se-SP inhibits OGD-induced neuronal toxicity. (A) Time-dependent cytotoxicity of OGD treatment on primary neurons. Primary neurons were cultured for 1–12 h under OGD condition. (B) Cytotoxicity of Se-SP or SP towards primary neurons. Primary neurons were treated with 5–20 μg/mL Se-SP or SP for 48 h. (C) Se-SP inhibits OGD-induced neuronal toxicity. Primary neurons were treated with 5 and 10 μg/mL Se-SP (or SP) or/and co-treated with OGD treatment for 6 h. Neural viability was determined using MTT assay. (D) Neuronal morphology. Morphology of primary neurons with or without tubulin staining was determined by phase contrast and fluorescence microscope (magnification, 200×). Bars with ‘*’ or ‘**’ represents *p* < 0.05 and *p* < 0.01, respectively. Bar with different letters means the statistic difference at *p <* 0.05.

Neuronal toxicity in OGD-treated primary neurons was firstly examined by MTT assay. As shown in Figure 2(A), neuronal viability under OGD conditions showed a significant decrease in a time-dependent manner. Neurons exposed to 5, 10 and 20 μg/mL Se-SP or SP alone for 48 h showed no significant changes of cell viability (Figure 2(B)). However, Se-SP co-treatment significantly suppressed OGD-induced neurotoxicity, as demonstrated by the improved neuronal viability (Figure 2(C)). SP co-treatment showed no significant protective potential. Neuronal morphology further confirmed this protective effect. As shown in Figure 2(D), the normal hippocampal neurons under phase contrast microscope showed interconnected nerve fibres, which extended to form dense neural networks. OGD treatment not only decreased neuron number but also caused morphological changes, such as neuron shrinkage, breakage of axons and loss of interconnection. However, Se-SP co-treatment completely blocked this morphological change in OGD-treated neurons. SP co-treatment had no ability to improved neuron morphology. Neurons exposed to SP or Se-SP alone showed no morphological change. Taken together, these results suggested that Se-SP after Se addition achieved enhanced effects in blocking OGD-induced neurotoxicity.

### Se-SP attenuates OGD-induced neuronal apoptosis

**Figure 3.**
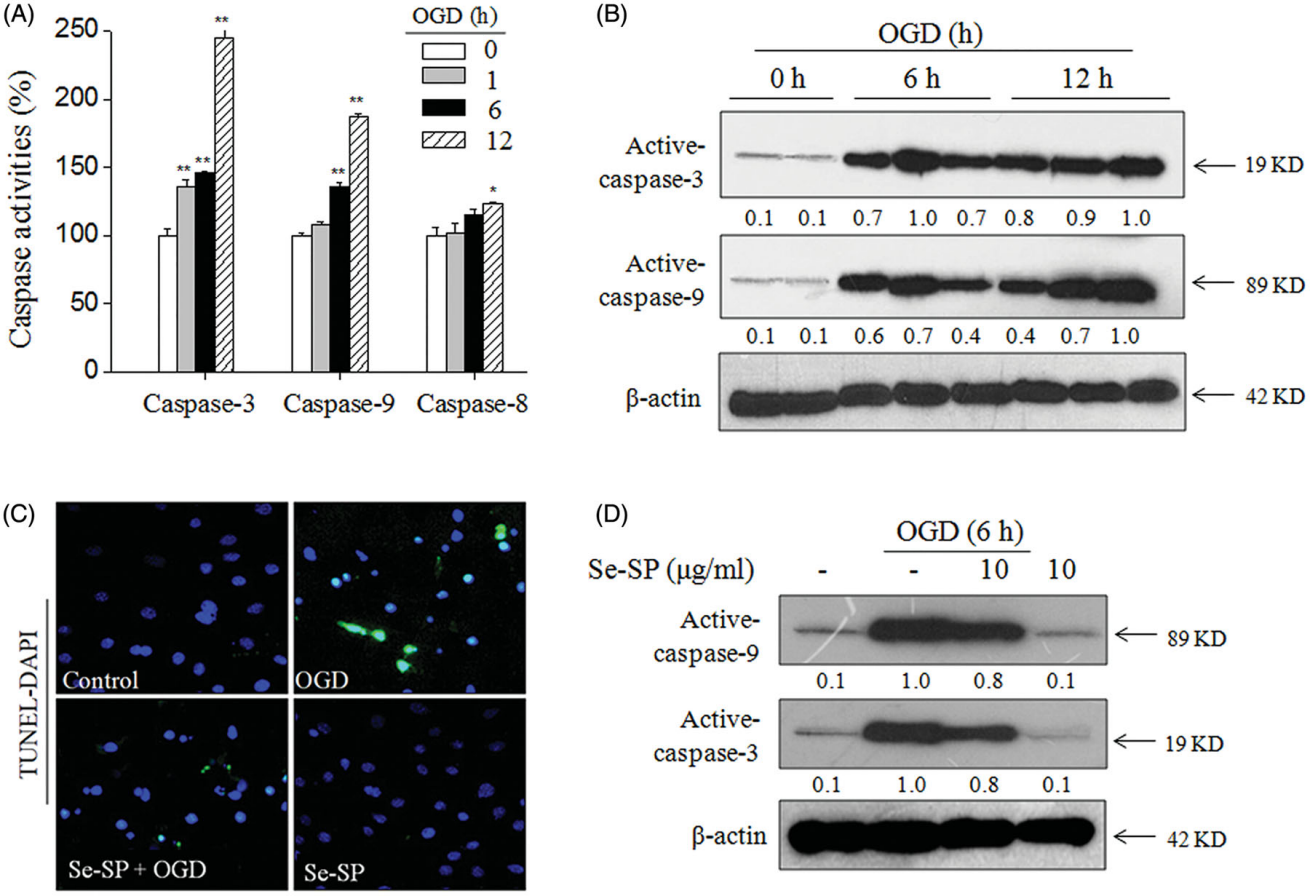
Se-SP attenuates OGD-induced neurons apoptosis. (A) Caspases activation induced by OGD. (B) Caspases expression induced by OGD. (C) TUNEL-DAPI staining. Nuclear condensation and DNA fragmentation were examined by TUNEL-DAPI double staining (magnification, ×200). (D) Se-SP attenuated OGD-induced caspases activation. Details of the experiments followed the methods part. Caspases activity and expression were measured by microplate reader and western blotting, respectively. Neurons apoptosis (TUNEL-positive) was determined by a fluorescence microscope. Bars with ‘*’ or ‘**’ suggests *p* < 0.05 and *p* < 0.01, respectively.

Apoptosis can be evoked by activating caspase-9 and caspase-8, which are the initiators of mitochondrial (intrinsic) and death receptor (extrinsic) signalling pathways (McIlwain et al. 2013), respectively. To explore the underlying mechanism by which OGD induced neurons apoptosis, the activity and expression of caspase-8, caspase-9 and downstream executioner caspase-3 were all examined. As shown in Figure 3(A), caspase-3, -8 and -9 in hippocampal neurons were significantly activated under OGD conditions. Caspase-9 activation was higher than that of caspase-8, indicating that OGD induced neuronal apoptosis mainly by triggering mitochondrial-mediated apoptosis pathways. Time-dependent caspases expression by western blot analysis (Figure 3(B)) and TUNEL-DAPI staining (Figure 3(C)) both further confirmed OGD-induced neuronal apoptosis. However, Se-SP co-treatment effectively attenuated OGD-induced neuronal apoptosis, as revealed by the decrease of TUNEL-positive cells (Figure 3(C)) and caspases expression (Figure 3(D)). Taken together, these results revealed that Se-SP had the potential to inhibit OGD-induced neuronal apoptosis.

### Se-SP improves OGD-induced mitochondrial dysfunction by modulating Bcl-2 family

**Figure 4.**
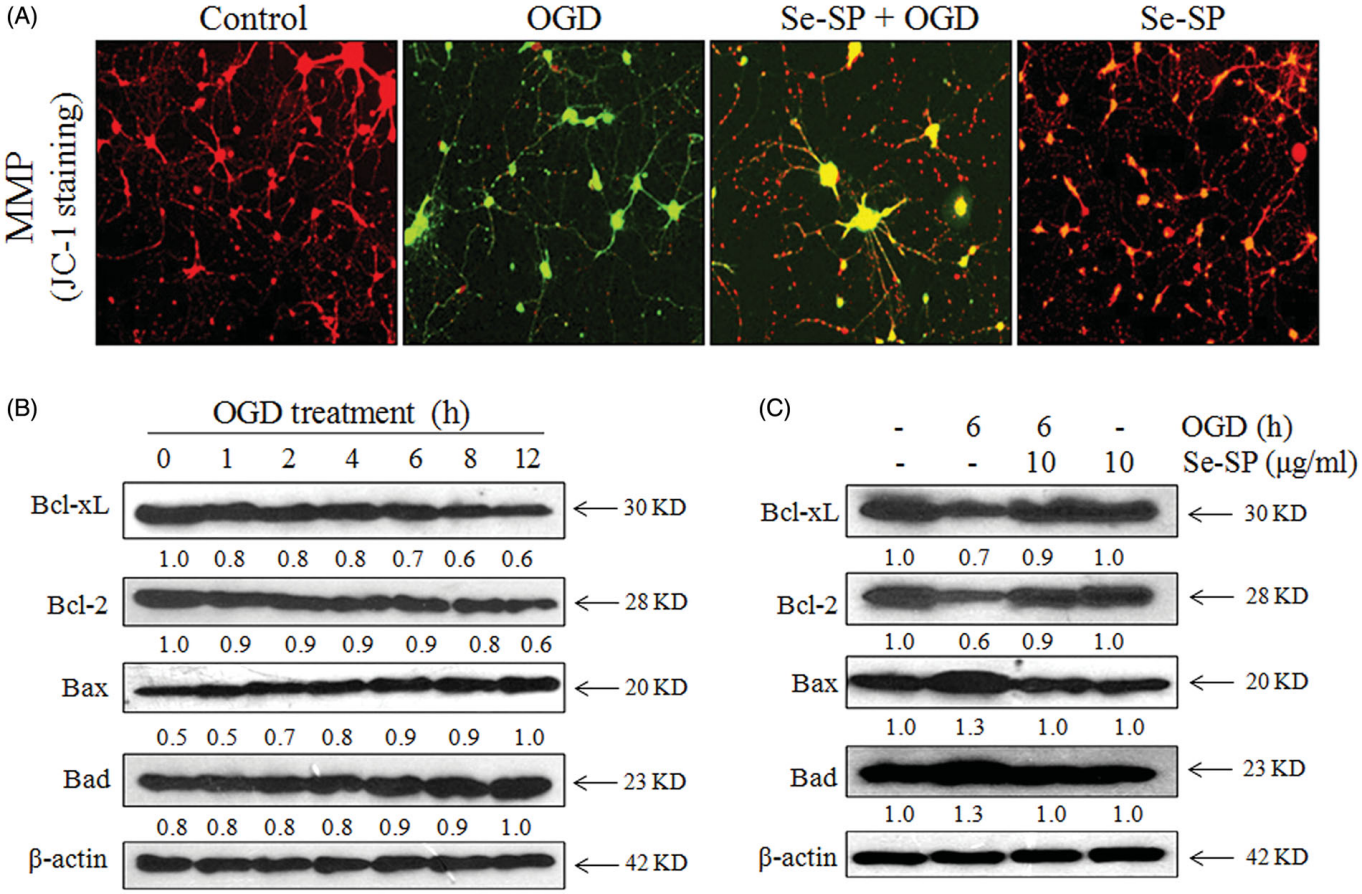
Se-SP improves OGD-induced mitochondrial dysfunction by regulating Bcl-2 family. (A) Se-SP prevented OGD-caused mitochondrial membrane potential dissipation. mitochondrial membrane potential (MPP) was detected by JC-1 probe as described in the method section. (B) Time-dependent dysfunction of Bcl-2 family in OGD-treated neurons. (C) Se-SP normalized Bcl-2 family proteins expression in OGD-treated neurons. Bcl-2 family expression was detected by western blotting.

Mitochondrial membrane potential (MMP) was examined by JC-1 probe, which gathers in mitochondrial matrix with red fluorescence. Meanwhile, JC-1 exists in the form of monomer with green fluorescence when MMP is low (depolarisation), which can be used to reflect the loss of MMP. As shown in Figure 4(A), OGD-injured hippocampal neurons showed enhanced green fluorescence, implying the loss of MMP in OGD-treated neurons. However, Se-SP co-treatment markedly blocked OGD-induced MPP loss, as reflected by the enhanced red fluorescence. SP co-treatment caused no significant change of MPP. Bcl-2 family proteins as the components of the outer mitochondrial membrane are involved in cell apoptotic death (Zheng et al. 2020). Hence, the expression of anti- (Bcl-2, Bcl-xL) and pro-apoptotic (Bax, Bad) proteins of Bcl-2 family in OGD-treated neurons were further examined by western blotting. The data revealed that Bcl-2 and Bcl-xL were time-dependently down-regulated in the OGD-treated primary hippocampal neurons. Bax and Bad were up-regulated in a time-dependent manner under OGD conditions (Figure 4(B)). However, Se-SP co-treatment significantly balanced Bcl-2 family expression in OGD-treated neurons (Figure 4(C)). Taken together, these results suggested that Se-SP had the potential to improve OGD-induced mitochondrial dysfunction by modulating Bcl-2 family.

### Se-SP suppresses OGD-induced oxidative damage through inhibiting ROS accumulation

**Figure 5.**
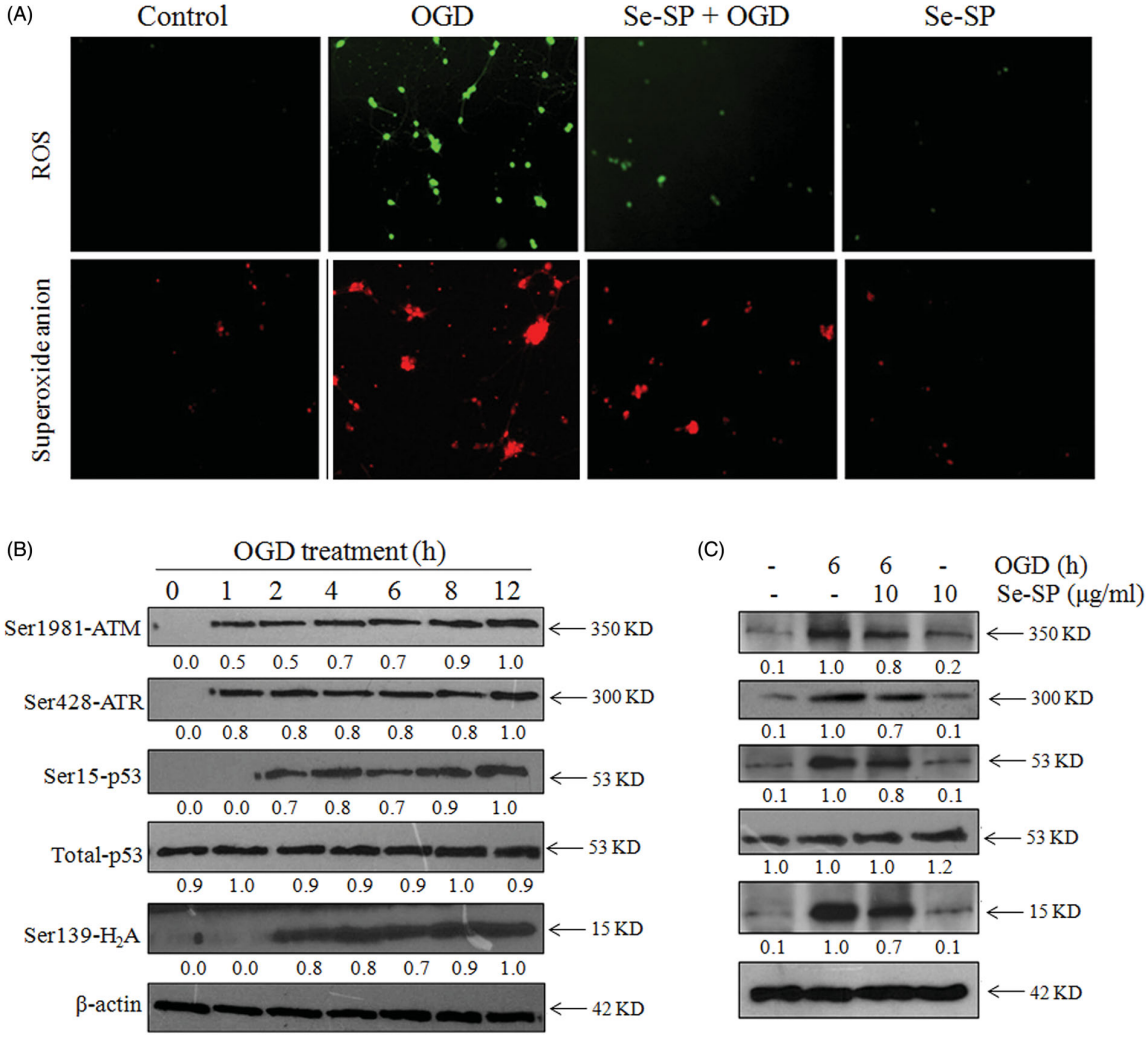
Se-SP suppresses OGD-induced oxidative damage by inhibiting ROS accumulation. (A) Se-SP prevented OGD-induced ROS accumulation. Primary neurons were loaded with DCFH-DA or Mito-SOX before treatment for detection of ROS and superoxide anion, respectively. Images were observed by a fluorescence microscope. (B) Time-dependent DNA damage in OGD-treated neurons. (C) Se-SP suppressed OGD-induced DNA damage. Details of the experiments followed the methods part. Protein expression was detected by western blotting method.

Reactive oxygen species (ROS)-mediated oxidative damage plays a critical role in the pathogenesis of ischaemic stroke (Rodrigo et al. 2013). Mitochondria as the main source of cellular ROS can produce a huge amount of superoxide and hydrogen peroxide under kinds of detrimental stimuli (Moro et al. 2005; Christophe and Nicolas 2006). Herein, two fluorescent probes, DCFH-DA and Mito-SOX Red, were respectively used to determine whether Se-SP makes an important difference in the production of intracellular ROS and intra-mitochondrial superoxide anion in OGD-induced neurons. The results showed that OGD-injured primary neurons exhibited enhanced green and red fluorescence, indicating the obvious overproduction of ROS and superoxide anion in OGD-treated neurons. However, Se-SP co-treatment significantly inhibited OGD-induced accumulation of ROS and superoxide anion (Figure 5(A)). Se-SP alone caused no significant generation of ROS and superoxide anion. ROS is known to cause intracellular molecule damage and even trigger cell apoptosis. Therefore, western blotting was employed to investigate OGD-mediated DNA damage. As shown in Figure 5(B), OGD treatment caused time-dependent phosphorylation of Ser1981-ATM, Ser428-ATR, Ser139-H2A, Ser15-p53. However, Se-SP co-treatment significantly repressed OGD-induced DNA damage, as demonstrated by the decreased phosphorylation level (Figure 5(C)). Taken together, these results indicated that Se-SP had the potential to suppress OGD-induced oxidative damage via inhibiting ROS generation.

### Inhibition of MPTP blocks OGD-induced neurotoxicity

**Figure 6.**
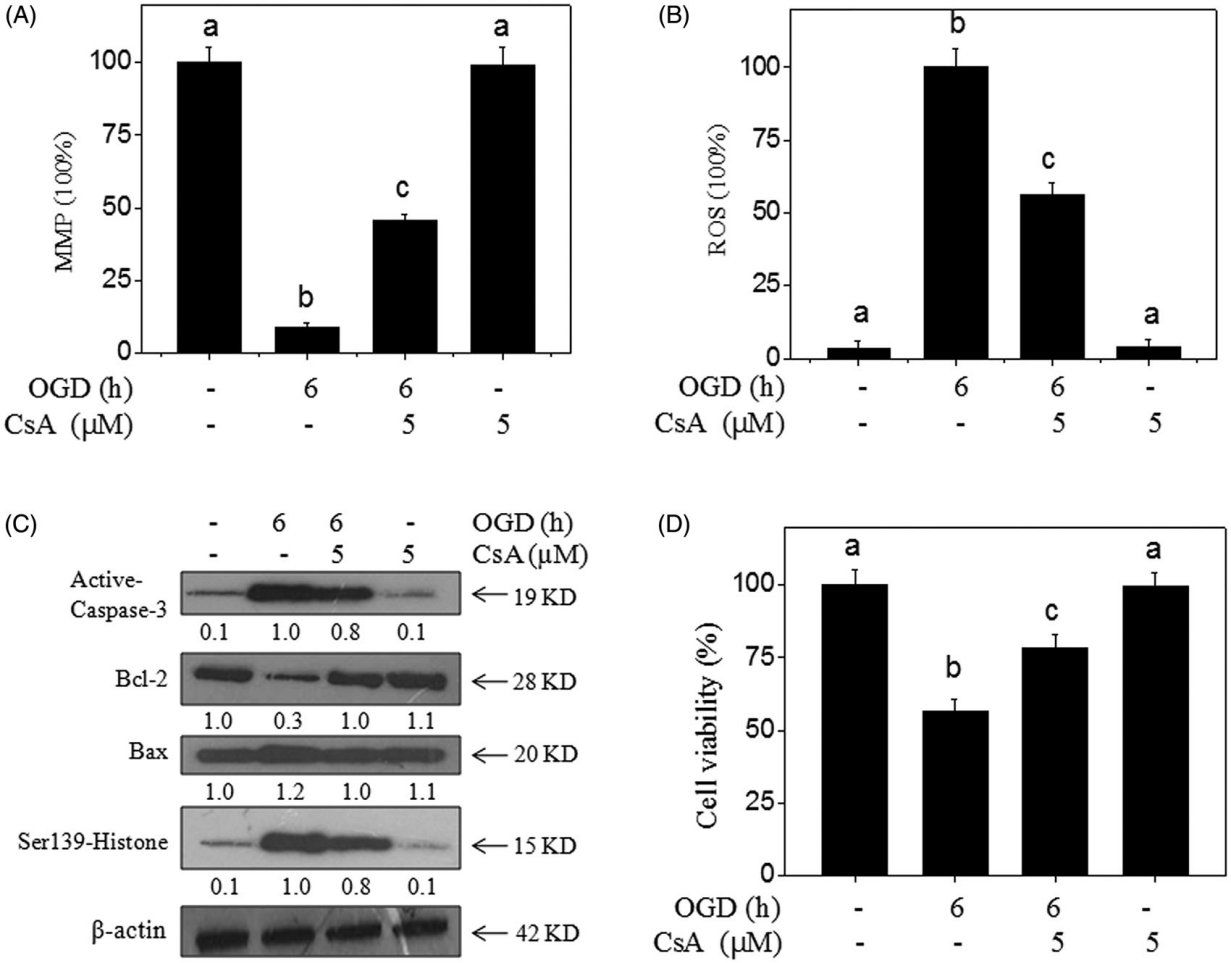
MPTP inhibition blocks OGD-induced neural toxicity. CsA (MPTP inhibitor) improved the MPP (A) and inhibited ROS generation (B) in OGD-treated neurons. Primary neurons were pre-treated with 5 μM CsA for 30 min and co-treated with OGD. (C) Effects of CsA on protein expression in OGD-treated neurons. Protein expression was detected by western blotting method. (D) MPTP inhibition blocks OGD-induced neural toxicity. Neural viability was examined by MTT assay. Bar with different letters means the statistic difference at *p <* 0.05.

Numerous studies have revealed that the opening of MPTP plays an important role in triggering ROS generation and mitochondrial apoptotic cascade (Andrabi et al. 2020). Herein, MPTP inhibitor (CsA) was used to further investigate the significant role of MPTP. The results indicated that MPTP inhibition by CsA significantly improved the MPP (Figure 6(A)) and inhibited ROS generation (Figure 6(B)) in OGD-treated neurons. Meanwhile, the western blotting results showed that MPTP inhibition effectively attenuated OGD-induced neuron apoptosis, DNA damage, and Bcl-2 family imbalance (Figure 6(C)), and eventually inhibited OGD-induced neurotoxicity (Figure 6(D)). Taken together, these results suggested that Se-SP had the potential to inhibit OGD-induced neurotoxicity and apoptosis by regulating MPTP opening.

## Discussion

Selenium with novel antioxidant potential has been verified to exert a neuroprotective effect on neurodegenerative diseases, cerebral traumatic injury and ischaemic damage (Naziroglu et al. 2014; Balaban et al. 2017; Wang et al. 2018). Due to the severe side effects, inorganic selenium and organic selenium have not been considered for application. Our preliminary test also confirmed that Na_2_SeO_3_ showed no protective effect on OGD-treated neurons, but caused significant toxicity to neurons. However, selenium-containing proteins as a new form of selenium display novel antioxidant activity, and showed no side effects. Hence, the transformation of inorganic selenium into selenium-containing proteins attracted much attention recently. Se was enzymatically or non-enzymatically metabolized in an organism, and Se was finally incorporated into the Se-containing peptides or Se-containing proteins. Selenocysteine (SeCys) was the main existing form in an organism. Herein, in the present study, Na_2_SeO_3_ as an inorganic selenium species was used as a selenium source to produce selenium-containing protein in *S. platensis*. *Spirulina platensis* is a suitable carrier of selenium, and protein from Se-enriched *S. platensis* after Se merger has achieved augmented antioxidant capacity and was able to attenuate hepatocyte apoptosis, osteoblastic dysfunction and erythrocyte injury by inhibiting oxidative stress (Zhang et al. 2011; Fan et al. 2012; Sun et al. 2018). Moreover, Se-SP has been shown to prevent chemotherapeutic drug-induced osteocytotoxicity by alleviating mitochondrial dysfunction (Sun et al. 2018). However, whether Se-SP can protect neurons from oxidative stress-induced ischaemic injury has not been reported. Herein, this study revealed that Se-SP exhibits neuroprotective effects against OGD-induced neurotoxicity and apoptosis in primary hippocampal neurons by inhibiting ROS-mediated oxidative damage and mitochondrial dysfunction via regulating MPTP opening.

The pyramidal neurons in hippocampal CA1 region are vulnerable to ischaemic injury (Petito et al. 1987), and a large number of studies have explored that several molecular and cellular mechanisms associated with neuronal dysfunction were involved in ischaemic stroke (Yu et al. 2017; Wang et al. 2018). Hence, an *in vitro* OGD model of primary hippocampal neurons in rats was established in our experiment to simulate ischaemic conditions in stroke. ROS are critical players in OGD-induced brain injury (Rodrigo et al. 2013). As we all know, intracellular ROS is produced in many important biological processes including energy generation and cell signalling (Moro et al. 2005; Christophe and Nicolas 2006; Drose et al. 2016), and cells are capable of scavenging ROS through various enzymatic and nonenzymatic reactions to maintain the balance of cellular redox (Rodrigo et al. 2013). A considerable number of studies have indicated that mitochondria in ischaemic injury generate a large amount of ROS, including superoxide anion (O^2−^) and its derivative (hydrogen peroxide, hydroxyl radical, and peroxynitrite) (Christophe and Nicolas 2006). In agreement with previous studies, we also found that OGD provoked the excessive production of intracellular ROS and mitochondrial superoxide, as shown by the enhanced green and red fluorescence in DCFH-DA and Mito-SOX Red (DHE derivative) staining neurons. In addition, OGD injury leads to a decrease in antioxidant enzymes (Milanlioglu et al. 2016; Wang et al. 2019). Consequently, the balance between ROS generation and elimination is destroyed, resulting in ROS-mediated oxidative damage to cellular constituents, such as nucleic acid, membrane proteins and lipids (Zitnanova et al. 2016; Zhou et al. 2019), which is further confirmed by our findings that the markers of DNA damage in neurons (Ser1981-ATM, Ser428-ATR, Ser139-H2A, Ser15-p53 and total-p53) are markedly increased after OGD treatment. However, the administration of Se-SP significantly attenuates OGD-induced ROS generation and DNA damage in primary hippocampal neurons. Selenium was accepted as a key factor in regulating the active sites of many Se-containing antioxidant enzymes, such as glutathione peroxidase (Glu-Px) and thioredoxin reductase (TrxR). We speculated that Se in the form of Se-SP was enzymatically or nonenzymatically metabolised in cells, and finally was incorporated into the Se-containing peptides or Se-containing enzymes, such as Glu-Px and TrxR. Selenium supplement replenished the antioxidant ability of antioxidant enzymes, and eventually inhibited OGD-induced ROS generation and DNA damage.

It has been shown that mitochondria are important regulators in OGD-caused cell apoptosis or necrosis (Liang et al. 2013; Ji et al. 2014; Liu et al. 2016). Mitochondria are critical performers in maintaining intracellular ion homeostasis and cellular bioenergetics (Spat and Szanda 2017; Yang et al. 2018). The stimulation of OGD can cause intracellular Ca^2+^ overload and ROS accumulation (Liu et al. 2016), both of which trigger the opening of MPTP (Toledo et al. 2014). MPTP is a multi-protein complex channel that controls the exchange of material between the cytoplasm and mitochondria. The opening of MPTP leads to the release of cytochrome *c* from mitochondria, reduction of bioenergy, a decline of MMP (Liang et al. 2013; Ji et al. 2014). Moreover, the mitochondrial membrane lipids were oxidized by excessive ROS, which may also lead to the depolarization of MMP. Loss of MMP exacerbates the pathological opening of MPTP. The interaction between MPTP and MMP results in a vicious circle and irreversible mitochondrial damage. Additionally, it has been reported that the loss of MMP and the oxidation of mitochondrial inner membrane lipids further promote the generation of ROS through respiratory chain uncoupling (Koshkin and Greenberg 2002). The opening of MPTP contributes to the over-production of ROS, which aggravates the activation of MPTP (Zorov et al. 2014). Prolonged MPTP activation leads to the release of pro-apoptotic proteins including cytochrome *c* into the cytoplasm, where apoptosome complex is formed and caspase-9 and caspase-3 are activated (Hao et al. 2005; McIlwain et al. 2013). Then, activated caspase-3 provokes PARP cleavage and caused DNA damage and cell apoptosis. Recently, a study has suggested that OGD injury brings about morphological damage of neurons, which is the reduction of primitive synapses, shortening of neuronal axons and shrinkage of neurons (Yu et al. 2017). Importantly, studies showed that the activation of MPTP is related to axonal degeneration and axon degeneration can be delayed by CsA (Barrientos et al. 2011). In line with previous reports, we also found that OGD caused the collapse of MMP, activation of MPTP, up-regulation of caspase-9 and caspase-3, DNA cleavage, an increase in neurons apoptosis and neuronal morphological changes, which were all effectively suppressed by Se-SP co-treatment. Additionally, ROS accumulation, MMP reduction, DNA damage, caspase-3 and Bax expression stimulated by OGD were as well attenuated by MPTP inhibitor (CsA) pre-treatment. Interestingly, the effect of CsA is similar to that of Se-SP. Our observations demonstrate that (1) mitochondrial ROS accumulation can stimulate MPTP opening, which activates the mitochondria-mediated signalling pathway, (2) mitochondrial-dependent apoptotic pathway may exert decisive effect on OGD-induced neurotoxicity, and (3) Se-SP can suppress the activation of mitochondrial dependent apoptotic pathway through preventing ROS generation and modulating MPTP opening. These data indicate that Se-SP plays a neuroprotective role by improving ROS-induced mitochondrial dysfunction and reducing neuron apoptosis.

Bcl-2 family proteins include anti-apoptotic and pro-apoptotic proteins, which are components of mitochondrial membrane pores and have been proved to be pivotal in mitochondria-mediated signal transduction pathway (Liang et al. 2013; Liu et al. 2016; Zheng et al. 2020). Under several pathological stimuli, such as ischaemic injury, pro-apoptotic proteins like Bax translocate to the outer mitochondrial membrane and form homo-oligomers (Hetz et al. 2005), which causes the increase of mitochondria membrane permeability and release of cytochrome *c*, which activates caspase-9 and subsequently other downstream proteins (Shimizu et al. 1999; Hetz et al. 2005). Finally, pro-apoptotic proteins evoked cascade event brings about cell apoptosis. However, this event can be prevented by Bcl-2 through binding to Bax and forming heterodimer (Bcl-2/Bax) (Yang et al. 1995; Adams and Cory 1998). Therefore, cell survival or apoptosis can be predicted by the ratio of Bcl-2/Bax. In the current study, we observed that OGD treatment caused a decrease in the ratio of Bcl-2 family anti-/pro-apoptotic proteins, as shown by western blot analysis. These alterations in OGD-injured neurons were attenuated by the addition of Se-SP, suggesting that Se-SP can exert an anti-apoptosis effect through balancing the Bcl-2 family.

## Conclusions

The present study demonstrated that Se-SP suppressed OGD-induced injury of primary hippocampal neurons *in vitro* through attenuating ROS-mediated DNA damage, mitochondrial dysfunction and apoptosis by regulating the opening of MPTP, which provided evidence that Se-SP is an effective novel drug for the treatment of ischaemic related neurological diseases, especially ischaemic stroke.
